# Vegetation Classification and Distribution Patterns in the South Slope of Yarlung Zangbo Grand Canyon National Nature Reserve, Eastern Himalayas

**DOI:** 10.3390/plants11091194

**Published:** 2022-04-28

**Authors:** Po-Po Wu, Zi Wang, Ning-Xia Jia, Shao-Qiong Dong, Xiao-Yun Qu, Xian-Guo Qiao, Chang-Cheng Liu, Ke Guo

**Affiliations:** 1 State Key Laboratory of Vegetation and Environmental Change, Institute of Botany, Chinese Academy of Sciences, Beijing 100093, China; wupopo@ibcas.ac.cn (P.-P.W.); wangzi@ibcas.ac.cn (Z.W.); jianingxia@ibcas.ac.cn (N.-X.J.); dongshaoqiong21@mails.ucas.ac.cn (S.-Q.D.); quxiaoyun21@mails.ucas.ac.cn (X.-Y.Q.); qiaoxianguo@ibcas.ac.cn (X.-G.Q.); 2 University of Chinese Academy of Sciences, Beijing 100049, China

**Keywords:** vertical vegetation belts, vegetation classification, Himalayas, biodiversity

## Abstract

Yarlung Zangbo Grand Canyon National Nature Reserve has the most complete vertical vegetation belts in China. However, identification and distribution of vertical vegetation belts is still uncertain and in debate. To explore the above issues, 190 plots were surveyed within the reserve from 2019 to 2021. Based on the vegetation plot data, cluster analysis, ordination analysis, and biodiversity statistics were performed to reveal the structure of vertical vegetation belts–the driving factors of vegetation distribution–to describe the main biodiversity patterns. Five vertical vegetation belts were identified by clustering. NMDS ordination showed that the main factor of vegetation distribution is elevation. Based on the results of the analysis and previous literature, a new scheme of vertical vegetation belts in the south slope of the reserve was proposed. There was a lower montane seasonal rainforest belt (600–1100 m), a lower montane evergreen broadleaf forest belt (1100–1800 m), a middle montane semi-evergreen broadleaf forest belt (1800–2400 m), a subalpine evergreen needleleaf forest belt (2400–3800 m), a alpine shrubland and meadow belt (3800–4400 m), an alpine sparse vegetation belt (4400–4800 m), and a nival belt (4800–7782 m). Among them, the seasonal rainforest belts are the northernmost distribution of this type, and the semi-evergreen broadleaf forest belts exist only in the Eastern Himalayas. The study showed a unimodal pattern in plant species diversity, the peak of which is about 1900 m. The middle montane semi-evergreen broadleaf forest belt had the highest species diversity in the reserve. This study settled the issues regarding the vertical vegetation belts, the main drivers of vegetation and assessment of plant species diversity in the south slope of the Yarlung Zangbo Grand Canyon National Nature Reserve. It provides essential support for the management and conservation of these ecosystems in the reserve.

## 1. Introduction

Recognizing and using elevational subdivisions is at the core of biogeographical and ecological studies in mountain ecosystems [1]. One of their key research areas is the recognition and use of vertical vegetation belts. The study of Andean vertical vegetation belts by Humboldt and Bonpland is considered the first work [2]. According to indicator species and elevations, Andean vegetation was divided into seven vertical belts. After more than two hundred years, the record of vertical belts provides important evidence for the relationship between the rising of the vertical vegetation belt and global climate change [3]. The studies of vertical vegetation belts were carried out in the Rocky Mountains, Alps, Mount Kilimanjaro, and other mountain ranges [4,5,6,7,8]. Due to the limitation of latitude, climatic zone, and elevation span, their vertical vegetation belts are relatively simple. In the Alps, for example, the base belt is a deciduous broadleaf forest [6]. Such as with Mount Kilimanjaro, located near the equator, its base belt is savanna. Due to the extent of the elevational gradient, suitable latitude, and southwest monsoon, the Himalayas have abundant vertical vegetation belts and are an excellent place for testing macroecological and biogeographical hypotheses.

The Yarlung Zangbo Grand canyon, located in the Eastern Himalayas, has the most complete vertical vegetation belts in China, and is one of the most complete vertical vegetation belts in the world [9]. In addition, it is one of the deepest and longest canyons in the world, as well as the most important passageway of water vapor transported from the Indian Oceans to the Qinghai-Tibet Plateau [10,11,12]. The Mount Namjagbarwa above this canyon is 7782 m above sea level, which is the highest peak in the Eastern Himalayas. The abundant water vapor coupled with a great elevational gradient, together, create the diverse vertical vegetation belts. At the elevational gradient of more than 7000 m, the vertical vegetation belts gradually transit from the tropical seasonal rainforest to the alpine sparse vegetation and permanent snow [9,13]. The vertical vegetation belts are complete and unique. The tropical seasonal rainforest belt here is located at 29 degrees 37 s, north latitude, which is the northernmost tropical seasonal rainforest in the world [14,15]. In addition, there is a distinctive semi-evergreen broadleaf forest belt, which exists only in the Eastern Himalayas.

The Yarlung Zangbo Grand canyon is also one of the global biodiversity hotspots [16,17]. Complete vertical vegetation belts have diverse and complex ecosystems which provide diverse habitats for a large number of species. There are more than 3800 vascular plant species in an area of 9168 km^2^ [18]. In recent years, many new species of animals and plants are continuously discovered in the canyon [19,20,21,22,23,24,25,26,27]. For example, Medog County, located in the canyon, is the county with the largest number of new species discovered in China in 2020 [28]. In order to protect the water vapor channel, vertical vegetation belts and biodiversity, Yarlung Zangbo Grand Canyon is designated as a national nature reserve.

However, vegetation surveys and research in Yarlung Zangbo Grand Canyon National Nature Reserve are hampered by the complicated environments and inconvenient transportation. It was until 1980 that Li et al. systematically reported the vegetation here for the first time, based on fifteen months of fieldwork [9,13]. Vegetation on the south slope of the mountain is divided into eight vertical vegetation belts: one, a lower mountain evergreen monsoon rainforest belt (below 600 m); two, a lower mountain semi-evergreen monsoon rainforest belt (600–1100 m); three, a middle mountain evergreen broadleaf forest belt (1100–1800 m); four, a middle mountain semi-evergreen broadleaf forest belt (1800–2400 m); five, a subalpine hemlock forest belt (2400–2800 m); six, a subalpine fir forest belt (2800–4000 m); seven, an alpine shrubland and meadow belt (4000–4400 m); eight, an alpine subnival vegetation belt (4400–4800 m). Other researchers also report different classifications schemes of vertical vegetation belts in the region [29,30,31]. However, these studies are mainly based on descriptive materials and expert experiences and opinions. The vertical vegetation belts in Yarlung Zangbo Grand Canyon National Nature Reserve are still controversial due to the lack of quantitative analysis based on vegetation plots data, such as the validity of the semi-evergreen broadleaf forest and broadleaved mixed forest belts, which need to be verified. Different vertical vegetation belts were defined within the same elevation range [9,29,30,31]. In order to resolve these disputes, more quantitative vegetation studies are needed.

The purpose of this study is to identify main vertical vegetation belts, the main environmental drivers of vegetation distribution, as well as to assess composition of plant communities and plant species diversity in the south slope of Yarlung Zangbo Grand Canyon National Nature Reserve. There are notable conservation gaps for disturbance of future climate change on ecosystem functioning and services [32], which require vegetation data to provide the necessary guidance for ecosystem conservation [33,34,35,36]. Based on the results of statistical analysis of quantitative data, we hope that the study can improve the management of the reserve. Meanwhile, discovering its composition and biodiversity would contribute to protecting the habitats of endangered species and resolving the “Humboldt’s enigma”.

## 2. Results

### 2.1. Vertical Vegetation Belts

**Figure 1:**
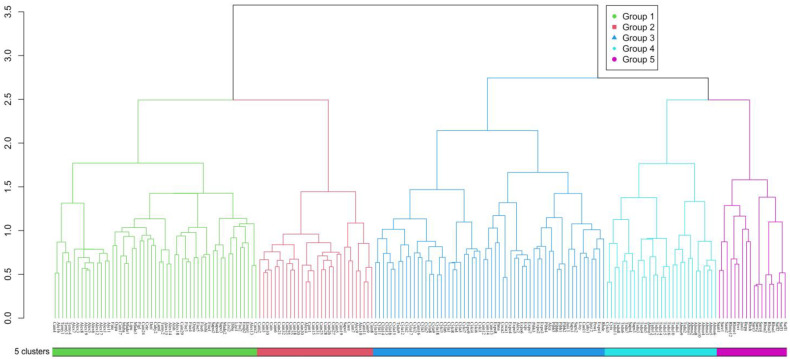
Cluster dendrogram of the 190 plots in the south slope of Yarlung Zangbo Grand Canyon National Nature Reserve.

**Figure 2:**
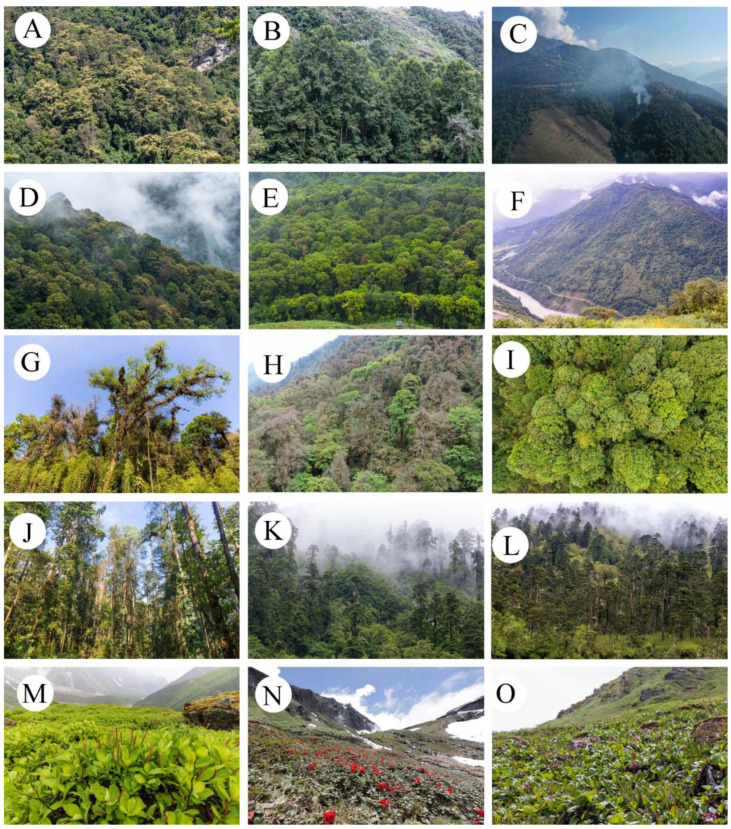
Typical alliances of five vertical vegetation belts. (**A**–**C**), lower montane seasonal rainforest belt; (**A**), *Terminalia myriocarpa* forest alliance; (**B**), *Altingia excels* forest alliance; (**C**), the “slash-and-burn” farming method; (**D**–**F**), lower montane evergreen broadleaf forest belt; (**D**), *Castanopsis indica* forest alliance; (**E**), *Castanopsis ceratacantha* forest alliance; (**F**), *Macaranga denticulate* forest alliance; (**G**–**J**), middle montane semi-evergreen broadleaf forest belt; (**G**), *Cyclobalanopsis kiukiangensis* forest alliance; (**H**), *Cyclobalanopsis lamellose* forest alliance (April); (**I**), *Exbucklandia populnea* forest alliance; (**J**), *Pinus bhutanica* forest alliance; (**K**,**L**), subalpine evergreen needleleaf forest belt; (**K**), *Tsuga dumosa* forest alliance; (**L**), *Abies delavayi* var. *motuoensis* forest alliance; (**M**–**O**), alpine shrubland and meadow belt; (**M**), *Salix annulifera* deciduous broadleaf shrubland alliance; (**N**), *Rhododendron chamaethomsonii* evergreen broadleaf shrubland alliance; (**O**), *Bergenia purpurascens* alpine meadow grassland alliance.

The vertical vegetation belts were identified by ward’s clustering and indicator species analysis. The clustering of all the 190 plots in the south slope of Yarlung Zangbo Grand Canyon National Nature Reserve produced five different groups by defining a K value = 5, based on fusion level value and silhouette width (Figure 1). The figures of fusion level value and silhouette width are provided in Appendix A. These groups correspond to five different vertical vegetation belts, respectively. There was a lower montane seasonal rainforest belt, a lower montane evergreen broadleaf forest belt, a middle montane semi-evergreen broadleaf forest belt, a subalpine evergreen needleleaf forest belt, and an alpine shrubland and meadow belt. In the five vertical vegetation belts, the top 20 indicator species of each belt are summarized in Appendix B. In addition, photos of typical alliances of each vertical belt are provided in Figure 1. The features of typical alliances in each vertical vegetation belt are also described in Figure 2.

Figure 2A–C, lower mountain seasonal rainforest belt (600–1100 m). Figure 2A, *Terminalia myriocarpa* forest alliance. The mean cover of the alliance is 70–80%. The mean height is 30–40 m. The community structure can be divided into tree layer, shrub layer and herb layer. In some primitive forests, the epiphytes and lianas are well developed. In tree layer, *Terminalia myriocarpa* is the dominant species and usually has plank buttresses root. Common species mainly are *Garcinia pedunculata*, *Cordia dichotoma*, *Gynocardia odorata*, *Homalium ceylanicum*, *Syzygium balsameum*, *Turpinia pomifera* and *Talauma hodgsonii*. In shrub layer, *Dendrocnide sinuate*, *Boehmeria macrophylla* var. *rotundifolia*, *Glochidion hirsutum*, *Ficus heteropleura* and *Rhynchotechum ellipticum* are common species. In herb layer, *Phrynium placentarium*, *Curculigo capitulate*, *Nephrolepis cordifolia*, *Pteris wallichiana* and *Pronephrium medogensis* are common species. Epiphytes and lianas mainly are *Neottopteris nidus*, *Lysionotus serratus*, *Lemmaphyllum drymoglossoides*, *Tetrastigma hypoglaucum*, *Rhaphidophora luchunensis* and *Rhaphidophora decursiva.* Figure 2B, Altingia excels forest alliance. The mean cover of the alliance is 80–90%. The mean height is 20–25 m. As the trunk of *Altingia excels* is white, this alliance was protected as fengshui forest near the village. But shrub layer and herb layer were more damaged. The community structure can be divided into tree layer, shrub layer and herb layer. In tree layer, *Altingia excels* is the dominant species. Common species mainly are *Meliosma pinnata*, *Brassaiopsis hainla*, *Albizia sherriffii* and *Macaranga denticulate*. In shrub layer, *Oxyspora paniculata*, *Saurauia griffithii*, *Maesa montana*, *Glochidion hirsutum* and *Buddleja myriantha* are common species. In herb layer, *Nephrolepis cordifolia*, *Amischotolype hispida*, *Selaginella effuse*, *Impatiens namchabarwensis* and *Elatostema hookerianum* are common species. Epiphytes and lianas mainly are *Millettia pachycarpa*, *Pothos chinensis*, *Aeschynanthus stenosepalus*, *Poikilospermum suaveolens* and *Dalbergia mimosoides*. Figure 2C,F, *Macaranga denticulate* forest alliance. The slash-and-burn farming method basically destroyed all the vegetation in the elevation range of 600–1900 m. After farmland was abandoned, secondary forest dominated by *Macaranga denticulate* was gradually formed. The forest has a simple structure and low species diversity. The mean cover of the alliance is 60–80%. The mean height is 22–28 m. Common species mainly are *Castanopsis indica*, *Saurauia punduana*, *Ficus semicordata*, *Oxyspora paniculata*, *Phrynium placentarium* and *Piper thomsonii*. Figure 2D–F, lower mountain evergreen broadleaf forest belt (1100–1800 m). Figure 2D, *Castanopsis indica* forest alliance. The mean cover of the alliance is 70–80%. The mean height is 22–28 m. The alliance is seriously disturbed by human activities. In tree layer, *Castanopsis indica* is the dominant species. Common species mainly are *Macaranga denticulate*, *Musa balbisiana*, *Saurauia napaulensis*, *Engelhardtia spicata*, *Ficus semicordata*, *Meliosma pinnata*, *Radermachera sinica*. In shrub layer, *Saurauia griffithii*, *Ardisia crenata*, *Glochidion hirsutum*, *Luculia pinceana* and *Maesa montana* are common species. In herb layer, *Phrynium placentarium*, *Pronephrium medogensis*, *Piper thomsonii*, *Curculigo capitulate*, *Nephrolepis cordifolia*, *Dicranopteris ampla*, *Impatiens namchabarwensis*, *Colocasia esculentum* var. *antiquorum* and *Elatostema acuminatum* are common species. Epiphytes and lianas mainly are *Embelia floribunda*, *Embelia parviflora*, *Dalbergia mimosoides*, *Smilax aspericaulis*, *Hedyotis scandens*, *Loxostigma griffithii*, *Lysionotus serratus* and *Lemmaphyllum drymoglossoides*. Figure 2E, *Castanopsis ceratacantha* forest alliance. The mean cover of the alliance is 80–90%. The mean height is 22–23 m. In tree layer, *Castanopsis ceratacantha* is the dominant species. Common species mainly are *Macaranga denticulate*, *Meliosma pinnata*, *Saurauia punduana*, *Eurya trichocarpa*, *Cyclobalanopsis kiukiangensis* and *Pyrenaria tibetana*. In shrub layer, *Coriaria nepalensis*, *Oxyspora paniculata*, *Dendrocalamus tibeticus*, *Pyrenaria tibetana* and *Ardisia crenata* are common species. In herb layer, *Rubus metoensis*, *Oplismenus compositus*, *Nephrolepis cordifolia*, *Impatiens nyimana*, *Onychium siliculosum* and *Carpesium abrotanoides* are common species. Epiphytes and lianas mainly are *Helixanthera terrestris*, *Piper petiolatum*, *Tripterospermum volubile* and *Neottopteris simonsiana*. Figure 2G–J, middle mountain semi-evergreen broadleaf forest belt (1800–2400 m). Figure 2G, *Cyclobalanopsis kiukiangensis* forest alliance. The mean cover of the alliance is 70–80%. The mean height is 35–45 m. The community structure can be divided into tree layer, shrub layer and herb layer. Primitive forests were well preserved, with well epiphytes and lianas. In tree layer, *Cyclobalanopsis kiukiangensis* is the dominant species and usually has plank buttresses root. In April and May, *Cyclobalanopsis kiukiangensis* shed all their leaves and quickly grow new ones in a dozen days. Common species mainly are *Cyclobalanopsis kiukiangensis*, *Cinnamomum iners*, *Cerasus conadenia*, *Sorbus medogensis*, *Rhododendron arboretum*, *Acer pectinatum*, *Toxicodendron wallichii* var. *microcarpum*, *Machilus duthiei*, *Helicia tibetensis*, *Styrax grandifloras*, *Ilex longecaudata* and *Elaeocarpus varunua*. In shrub layer, *Chimonocalamus tortuosus*, *Smilax myrtillus*, *Damnacanthus indicus*, *Edgeworthia gardneri*, *Viburnum sympodiale*, *Ficus neriifolia*, *Skimmia melanocarpa* and *Lasianthus chinensis* are common species. In herb layer, *Campylandra aurantiaca*, *Arisaema concinnum*, *Elatostema hookerianum*, *Elatostema medogense*, *Rubus fockeanus*, *Pilea anisophylla*, *Ophiorrhiza rosea*, *Hydrocotyle salwinica*, *Laportea bulbifera*, *Sarcopyramis nepalensis*, *Fagopyrum dibotrys*, *Galium hoffmeisteri*, *Monotropastrum humile*, *Arisaema erubescens* and *Disporum longistylum* are common species. Epiphytes and lianas mainly are *Uncaria scandens*, *Embelia parviflora*, *Tetrastigma serrulatum*, *Trachelospermum jasminoides*, *Piper petiolatum*, *Rhaphidophora luchunensis*, *Rhaphidophora decursiva*, *Hedera nepalensis* var. *sinensis*, *Holboellia latifolia*, *Haplopteris doniana*, *Polypodiodes amoena*, *Hymenophyllum simonsianum*, *Pholidota articulate*, *Remusatia vivipara*, *Agapetes forrestii*, *Agapetes praeclara*, *Aeschynanthus angustissimus*, *Thladiantha cordifolia*, *Pleione hookeriana* and *Dendrobium salaccense*. There are also more mosses and lichens in the forest. Its mean cover about is 20–30%. Figure 2H, *Cyclobalanopsis lamellose* forest alliance. This alliance is very similar to *Cyclobalanopsis kiukiangensis* forest alliance in community structure and species composition. Both of them share the same range of elevation and are the main type of middle mountain semi-evergreen broadleaf forest belt. Figure 2I, *Exbucklandia populnea* forest alliance. After forest of the belt was damaged, secondary forest dominated by *Exbucklandia populnea* was gradually formed. At the beginning of the succession, the alliance is small and dense with low species diversity. The mean cover of the alliance is 80–95%. The mean height is 15–25 m. In tree layer, Common species mainly are *Schima parviflora*, *Symplocos lucida*, *Pinus bhutanica*. Common shrubs and herbs mainly are *Myrsine semiserrata*, *Ternstroemia biangulipes*, *Daphne bholua*, *Ardisia garrettii*, *Calanthe brevicornu*, *Tricholepidium normale*, *Campylandra aurantiaca*, *Ainsliaea latifolia*. There are more litter on the ground, and its coverage about is 90%. Figure 2J, *Pinus bhutanica* forest alliance. On a degraded swamp, or bare ground after a landslide, secondary forest dominated by *Pinus bhutanica* was gradually formed. At the beginning of the succession, the alliance is small and sparse with low species diversity. But *Pinus bhutanica* grows very fast, it can grow more than 40 m high in about 60 years. The mean cover of the alliance is 50–80%. The mean height is 15–45 m. In tree layer, Common species mainly are *Gaultheria leucocarpa* var. *cumingiana*, *Ilex denticulate*, *Houpoea rostrate*, *Diploknema butyracea*, *Cyclobalanopsis lamellose*, *Exbucklandia populnea*, *Cinnamomum tamala*, *Elaeocarpus varunua* and *Alsophila spinulosa*. In shrub layer, *Viburnum erubescens*, *Enkianthus deflexus*, *Litsea cubeba*, *Rosa sericea*, *Ilex nothofagifolia*, *Calamus acanthospathus*, *Mycetia nepalensis*, *Lasianthus biermannii* and *Psychotria calocarpa* are common species. In herb layer, *Beccarinda tonkinensis*, *Ophiorrhiza rosea*, *Elatostema hookerianum*, *Sarcopyramis nepalensis*, *Elatostema nasutum*, *Pilea anisophylla* and *Balanophora harlandii* are common species. Epiphytes and lianas mainly are *Melocalamus elevatissimus*, *Rhaphidophora luchunensis*, *Rhaphidophora decursiva*, *Trachelospermum jasminoides*, *Hymenophyllum simonsianum*, *Neottopteris nidus*, *Pothos chinensis*, *Epigeneium rotundatum*, *Aeschynanthus lasiocalyx*, *Pyrrosia lanceolate*, *Odontochilus lanceolatus*, *Eria tenuicaulis*, *Bulbophyllum reptans*, *Dendrobium hookerianum*, *Pholidota articulate* and *Pyrrosia sheareri*. Figure 2K,L, subalpine evergreen coniferous forest belt (2400–3800 m). Figure 2K, *Tsuga dumosa* forest alliance. Tall and sparse primitive forests were well preserved. The mean cover of the alliance is 70–80%. The mean height is 35–45 m. The community structure can be divided into tree layer, shrub layer and herb layer. In tree layer, *Tsuga dumosa* is the dominant species. Common species mainly are *Gamblea ciliate* var. *evodiifolia*, *Acer campbellii*, *Helwingia japonica*, *Lindera obtusiloba* and *Euonymus frigidus*. In shrub layer, *Daphne bholua*, *Berberis wilsoniae*, *Neillia thyrsiflora*, *Edgeworthia gardneri*, *Decaisnea insignis*, *Ribes glaciale*, *Leycesteria formosa*, *Euonymus sanguineus* and *Rhododendron delavayi* are common species. In herb layer, *Oxalis leucolepis*, *Circaea alpine*, *Ainsliaea latifolia*, *Plagiogyria glauca*, *Epipogium aphyllum*, *Pilea anisophylla*, *Anaphalis margaritacea*, *Botrychium robustum*, *Hydrocotyle salwinica*, *Maianthemum fuscum*, *Arisaema wattii* and *Impatiens tenuibracteata* are common species. Epiphytes and lianas mainly are *Lepisorus scolopendrium*, *Coelogyne corymbosa*, *Pleione bulbocodioides*, *Hymenophyllum simonsianum*, *Agapetes praeclara*, *Aristolochia griffithii*, *Actinidia venosa*, *Schisandra rubriflora* and *Vaccinium dendrocharis*. There are also more mosses and lichens in the forest. Its mean cover about is 80–90%. Figure 2L, *Abies delavayi* var. *motuoensis* forest alliance. Tall and sparse primitive forests are well preserved. But near the forest line, the alliance becomes sparse and small. The mean cover of the alliance is 50–80%. The mean height is 15–45 m. The community structure can be divided into tree layer, shrub layer and herb layer. Lianas and epiphytes are almost absent. In tree layer, *Abies delavayi* var. *motuoensis* is the dominant species. Common species are *Gamblea ciliate* var. *evodiifolia*, *Acer campbellii*, *Sorbus wilsoniana*, *Daphniphyllum himalense* and *Betula utilis*. In shrub layer, *Fargesia melanostachys*, *Ribes glaciale*, *Lonicera tangutica*, *Enkianthus quinqueflorus*, *Hydrangea aspera* and *Clethra delavayi* are common species. In herb layer, *Sinopodophyllum hexandrum*, *Aletris pauciflora*, *Impatiens nyimana*, *Circaea alpine*, *Maianthemum atropurpureum*, *Synotis longipes*, *Arisaema elephas*, *Arisaema decipiens* and *Dryopteris wallichiana* are common species. Epiphytes and lianas mainly are *Haplopteris linearifolia*, *Rhododendron dendrocharis* and *Agapetes praeclara*. There are also more mosses and lichens in the forest. Its mean cover about is 80–90%. Figure 2M–O, alpine scrub and meadow belt (3800–4400 m). Figure 2M, *Salix annulifera* deciduous broadleaf shrubland alliance. The mean cover of the alliance is 70–90%. The mean height about is 0.2–0.4 m. In shrub layer, *Salix annulifera* is the dominant species. Common species are *Gaultheria trichophylla*, *Berberis taronensis*, *Cassiope selaginoides*, *Diplarche multiflora*, *Rhododendron viridescens* and *Aster albescens* var. *levissimus*. In herb layer, *Polygonum viviparum*, *Cremanthodium phyllodineum*, *Bergenia purpurascens*, *Potentilla leuconota* are common species. In winter, it will be covered by snow with a thickness of about 6 m. Figure 2N, *Rhododendron chamaethomsonii* evergreen broadleaf shrubland alliance. The mean cover of the alliance is 70–90%. The mean height about is 0.1–0.2 m. In shrub layer, *Rhododendron chamaethomsonii* which creeps and grows on the ground is the dominant species. Common species are *Diplarche multiflora*, *Rhododendron mekongense*, *Salix anticecrenata*, *Lonicera myrtillus* and *Gaultheria trichophylla*. In herb layer, *Polygonum viviparum*, *Cremanthodium rhodocephalum*, *Cardamine macrophylla*, *Saxifraga wardii* and *Saxifraga melanocentra* are common species. In winter, it will be covered by snow with a thickness of about 6 m. Figure 2O, *Bergenia purpurascens* alpine meadow grassland alliance. The mean cover of the alliance is 50–75%. The mean height about is 0.25 m. In herb layer, *Bergenia purpurascens* is the dominant species. Common species are *Cardamine macrophylla*, *Senecio lingianus*, *Dryopteris lepidopoda*, *Saussurea nimborum*. There are many large outcrops in the meadow. In winter, it will be covered by snow with a thickness of about 6 m.

#### 2.1.1. Lower Montane Seasonal Rainforest Belt (Group 1)

The elevation range of this belt was 600–1100 m. It lies at the base of the valley, which had experienced considerable slash-and-burn farming and longtime logging. Most of the primary vegetation had been destroyed, with some remnants in the valleys and steep slopes. There was a large area of secondary forests, but some saplings of dominant species from primary vegetation can be found in the underlayer. The top ten indicator species were *Altingia excels*, *Impatiens stenantha*, *Phrynium placentarium*, *Sambucus adnata*, *Impatiens namchabarwensis*, *Blumea balsamifera*, *Mussaenda decipiens*, *Terminalia myriocarpa*, *Boehmeria macrophylla* var. *rotundifolia*, and *Lagerstroemia minuticarpa*.

The *Terminalia myriocarpa* forest alliance, *Altingia excels* forest alliance, and *Lagerstroemia minuticarpa* forest alliance were the mainly primeval vegetation type. The secondary vegetation mainly included the *Ficus semicordata* forest alliance, *Macaranga denticulate* forest alliance, *Saurauia polyneura* var. *paucinervis* forest alliance, *Albizia sherriffii* forest alliance, *Castanopsis indica* forest alliance, *Castanopsis hystrix* forest alliance, *Dendrocalamus tibeticus* bamboo shrubland alliance, *Ostodes paniculata* forest alliance, *Oxyspora paniculata* evergreen broadleaf shrubland alliance, and *Musa balbisiana* shrubby grassland alliance. 

#### 2.1.2. Lower Montane Evergreen Broadleaf Forest Belt (Group 2)

The elevation range of this belt was 1100–1800 m. This vertical belt, which lies within the scope of human cultivation, had also experienced considerable destruction. The main reasons for primary forest destruction were slash-and-burn farming and longtime logging. The top ten indicator species were *Castanopsis indica*, *Glochidion hirsutum*, *Oplismenus compositus*, *Triumfetta cana*, *Desmodium sequax*, *Polygonum capitatum*, *Pteris cretica*, *Impatiens arguta*, *Colocasia antiquorum*, and *Strobilanthes dimorphotricha*.

The *Castanopsis indica* forest alliance, *Castanopsis hystrix* forest alliance, and *Castanopsis ceratacantha* forest alliance were the main primeval vegetation types. The secondary forest was similar to the lower montane seasonal rainforest belt. 

#### 2.1.3. Middle Montane Semi-Evergreen Broadleaf Forest Belt (Group 3)

The elevation range of this belt was 1800–2400 m. The top ten indicator species were *Cyclobalanopsis lamellose*, *Exbucklandia populnea*, *Pholidota articulata*, *Ficus sarmentosa*, *Damnacanthus indicus*, *Disporum bodinieri*, *Cyclobalanopsis kiukiangensis*, *Arisaema concinum*, *Vaccinium kingdom-wardii*, and *Myrsine semiserrata*.

The *Cyclobalanopsis lamellose* forest alliance and *Cyclobalanopsis kiukiangensis* forest alliance were the main primeval vegetation types. The *Exbucklandia populnea* forest alliance and *Pinus bhutanica* forest alliance were the main secondary forests. In addition, there were small contributions of the *Alcimandra cathcartii* forest alliance, *Salix psilostigma* forest alliance, *Juglans sigillata* forest alliance, and *Populus wilsonii* forest alliance. Usually, the *Cyclobalanopsis lamellose* forest alliance and *Cyclobalanopsis kiukiangensis* forest alliance were called the evergreen broadleaf forest. However, in the region, *Cyclobalanopsis lamellose* and *Cyclobalanopsis kiukiangensis* shed their leaves and grow new leaves within a month before the rainy season (April to May). Therefore, the alliances growing in Yarlung Zangbo Grand Canyon National Nature Reserve should be called a semi-evergreen broadleaf forest due to short time deciduous phenology. 

#### 2.1.4. Subalpine Evergreen Needleleaf Forest Belt (Group 4)

The elevation range of this belt was 2400–3800 m. The top ten indicator species were *Tsuga dumosa*, *Abies delavayi* var. *motuoensis*, *Acanthopanax evodiaefolius*, *Ribes glaciale*, *Acer campbellii*, *Pilea symmeria*, *Galium hoffmeisteri*, *Lindera obtusiloba* var. *heterophylla*, *Smilacina fusca*, and *Oxalis Leucolepis*. 

The main primeval vegetation types were the *Tsuga dumosa* forest alliance and *Abies delavayi* var. *motuoensis* forest alliance. The primeval vegetation in the range was subject to little human interference. In some plots, the average height of the dominant species was over 40 m and its average diameter at breast height was also over 1 m. However, above 3400 m, *Abies delavayi* var. *motuoensis* forests were often stunted by perennial avalanches. The thickness of the snow in the area in March can reach up to 6 m.

#### 2.1.5. Alpine Shrubland and Meadow Belt (Group 5)

The elevation range of this belt was 3800–4400 m. It was covered by snow for 6 months each year. There were frequent avalanches here that cause habitat fragmentation. Therefore, meadows and shrublands were mixed in the same vertical belt. The top ten indicator species were *Pleurospermum angelicoides*, *Dryopteris barbigera*, *Viola biflora*, *Athyrium attenuatum*, *Geranium polyanthes*, *Cardamine macrophylla*, *Polygonum polystachyum*, *Pedicularis lineata*, *Rosa taronensis*, and *Rhododendron viridescens*. 

The main primeval vegetation types were the *Rhododendron chamaethomsonii* evergreen broadleaf shrubland alliance, *Rhododendron viridescens* evergreen broadleaf shrubland alliance, *Rhododendron pumilum* evergreen broadleaf shrubland alliance, *Salix annulifera* deciduous broadleaf shrubland alliance, *Salix flabellaris* deciduous broadleaf shrubland alliance, *Salix rehderiana* deciduous broadleaf shrubland alliance, *Bergenia purpurascens* alpine meadow grassland alliance, *Polygonum viviparum* alpine meadow grassland alliance, and *Polygonum macrophyllum* alpine meadow grassland alliance.

### 2.2. Ordination of Vegetation

**Figure 3:**
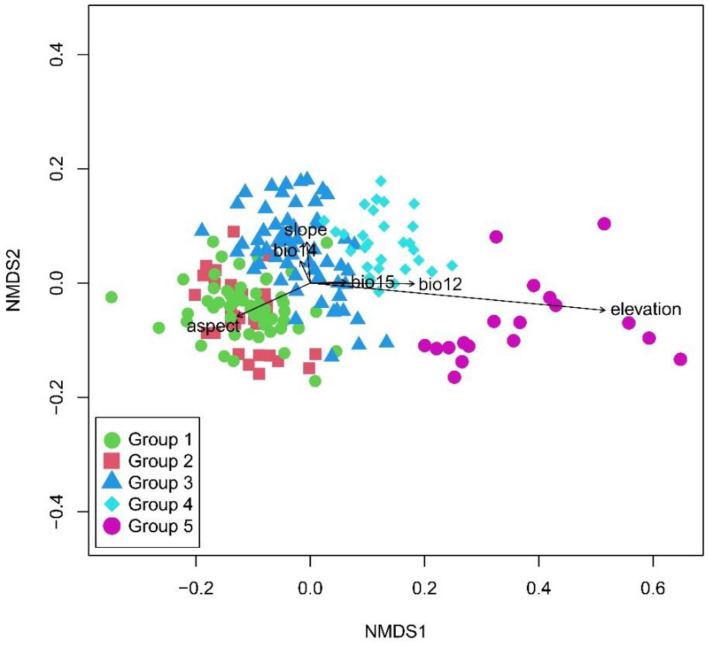
NMDS ordination of 190 vegetation plots showing differences in species composition between five vegetation belts identified based on clustering analysis with passively fitted environmental variables presented as arrows. Abbreviations: Bio12 = annual precipitation, Bio14 = precipitation of driest month, and Bio15 = precipitation seasonality.

**Figure 4:**
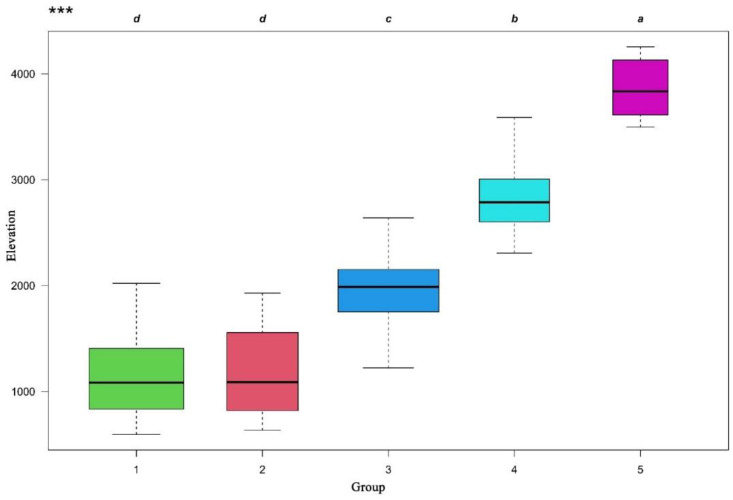
Boxplots presenting differences in elevation between five groups of vegetation belts evaluated by ANOVA and post-hoc Tukey test. Groups with the same letter did not differ significantly at *p* = 0.05. *** *p* ≤ 0.001.

The joint ordination diagram was obtained through overlaying the classification results onto the NMDS diagram (Figure 3). The NMDS ordination showed significant differences of the five vertical vegetation belts and their relationship with environmental factors (Table 1). The plots representing the five vertical belts were clearly separated into distinct groups, except with partial overlaps between group 1 and group 2. The first axis was mainly related to elevation. From left to right of the diagram, the elevation gradually increased, and the vegetation gradually changed from the lower montane seasonal rainforest belt, the lower montane evergreen broadleaf forest belt, the middle montane semi-evergreen broadleaf forest belt, and the subalpine evergreen needleleaf forest belt to alpine shrubland and meadow belt. The elevation range of each vegetation belt was showed in the boxplot (Figure 4). The lower montane seasonal rainforest belt and lower montane evergreen broadleaf forest belt had a similar elevation range. The post-hoc Tukey test between five groups was showed in Table 2. It showed the similarity of elevation ranges between group 1 and group 2.

### 2.3. Species Diversity

In the reserve, 1416 vascular plants from 190 plots were recorded, belonging to 165 families and 609 genera. Angiosperms included 136 families, 543 genera, and 1273 species; Gymnosperms included 3 families, 6 genera, and 10 species; Pteridophytes included 26 families, 60 genera, and 133 species. The family with the highest number of species was Orchidaceae, including 35 genera and 81 species.

**Figure 5:**
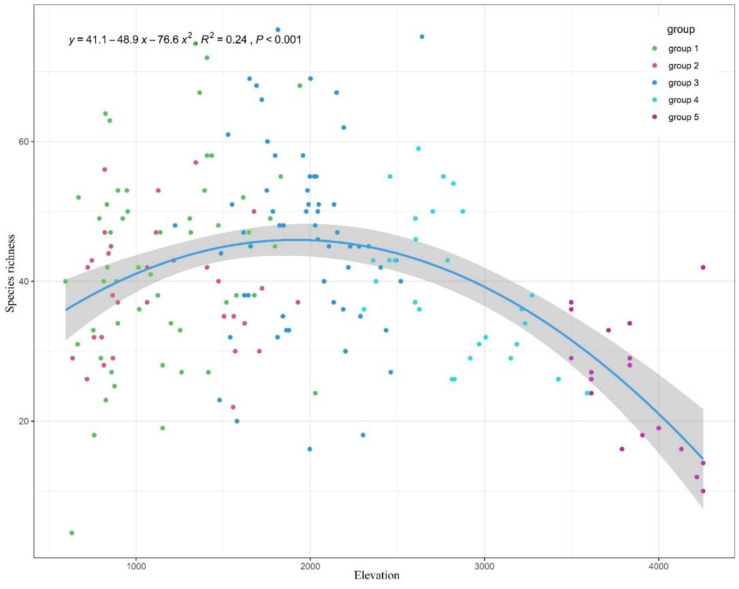
The relationship between species richness and elevation in the south slope of Yarlung Zangbo Grand Canyon National Nature Reserve.

The species richness, Shannon diversity index, Simpson diversity index, and Pielou diversity index were compared among five groups (Table 3). The maximum value of species richness was group 3. A unimodal pattern was showed in the scatter diagram between species richness and elevation (Figure 5), peaking at 1900–2000 m. Both of them showed that the middle montane semi-evergreen broadleaf forest belt had the highest biodiversity. There were 823 vascular plants recorded in the belt. 

### 2.4. The New Division Scheme of Vertical Vegetation Belts

**Figure 6:**
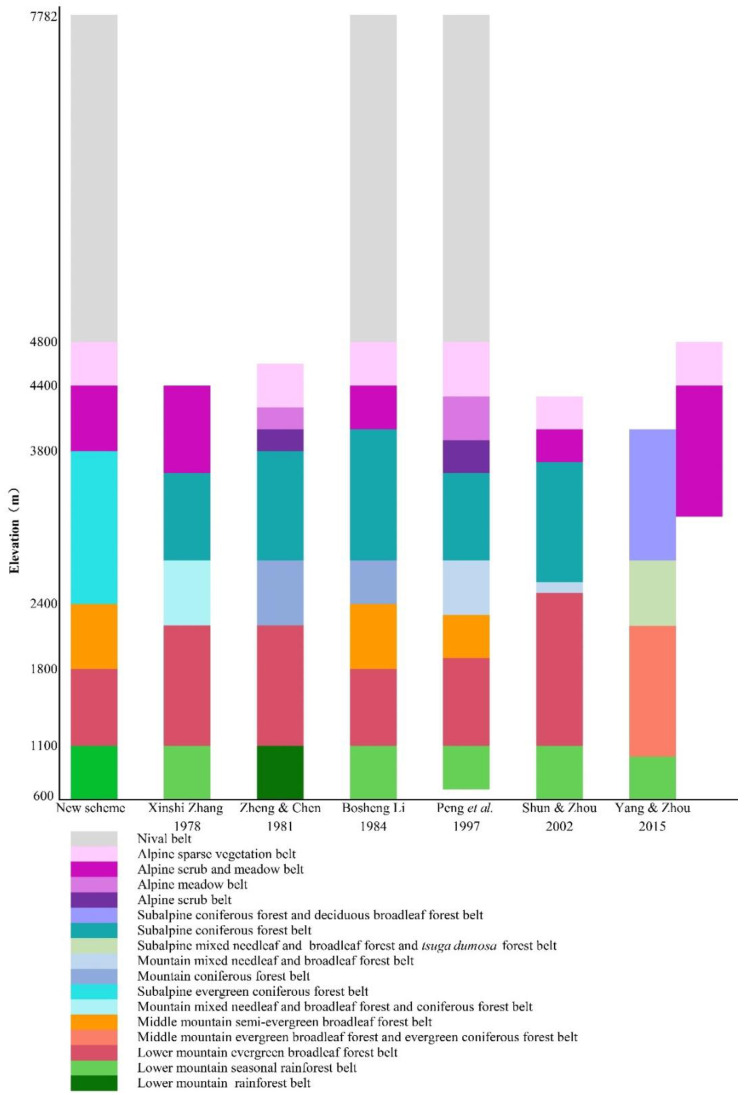
Comparison of different distribution schemes of vegetation belts in the study site.

Based on these results and previous literature, we proposed a new scheme for vertical vegetation belts in Yarlung Zangbo Grand Canyon National Nature Reserve. There were seven vertical vegetation belts: the lower montane seasonal rainforest belt (600–1100 m), lower montane evergreen broadleaf forest belt (1100–1800 m), middle montane semi-evergreen broadleaf forest belt (1800–2400 m), subalpine evergreen needleleaf forest belt (2400–3800 m), alpine shrubland and meadow belt (3800–4400 m), alpine sparse vegetation belt (4400–4800 m), and nival belt (4800–7782 m). This scheme was similar to the previous schemes in the lower montane seasonal rainforest belt, alpine shrubland and meadow belt, alpine sparse vegetation belt, and nival belt, but different in the lower montane evergreen broadleaf forest belt, middle montane semi-evergreen broadleaf forest belt, and subalpine evergreen needleleaf forest belt (Figure 6).

## 3. Discussion

### 3.1. Comparison of Vegetation Belts Distribution Schemes

The lower montane evergreen broadleaf forest belt and middle montane semi-evergreen broadleaf forest belt are considered as montane evergreen broadleaf forest belt in the studies of Xinshi Zhang, Du Zheng and Weilie Chen, and Hang Sun and Zhekun Zhou [29,30,37]. The main reason would be that the most important dominant species of two belts, both the *Castanopsis* and *Cyclobalanopsis* species, are considered as evergreen broadleaf species [38]. In addition, the vegetation plot data and physiognomic and phenological data are also insufficient. Therefore, the middle montane semi-evergreen broadleaf forest is doubted since it was first put forward [39]. Based on plots data, our quantitative analyses provided strong evidence for the validity of semi-evergreen broadleaf forests. There were large variations in species composition between the two vertical belts. Furthermore, the dominant species of the semi-evergreen broadleaf forest belt, *Cyclobalanopsis lamellose* and *C. kiukiangensis*, had a special deciduous phenological period, which was remarkably different from other *Cyclobalanopsis* species that dominated the evergreen forests in the subtropical region of eastern China. The special seasonal variation had been observed from 2019 to 2021 (Figure 2G,H). Most of the year, the physiognomy of this belt was evergreen, but during the deciduous period between April and May, the forest was brown because the tree layer shed all leaves in the dozen days before the rain season came. From May to June, the appearance of the forest changed from brown to red because the new leaves were red or brown-red and turned to green again in July. More detailed studies on the ecological and physiological adaptive mechanisms of these species to their environments are needed to explaining this distinct phenology.

In previous studies, the elevation range of 2400–2800 m is considered to be the subalpine hemlock forest belt, needleleaf and broadleaf mixed forest belt, or part of the evergreen needleleaf forest belt [9,13,29,31]. The main reason for the difference was that the division is based on their own experiences, which are limited by the scope of investigation and personal knowledge base at that time. Based on the vegetation plots data, our study showed that 2400–3800 m should be considered as subalpine evergreen needleleaf forest belt, which includes the subalpine hemlock forest sub-belt (2400–2800 m) and subalpine fir forest sub-belt (2800–3800 m).

The lower montane seasonal rainforest belt and lower montane evergreen broadleaf forest belts can be identified by clustering analysis. However, the NMDS ordination showed that there are high compositional similarities among these vegetation plots. The boxplots also showed large similarities in the elevation range. The main reason was that slash-and-burn farming and longtime logging have destroyed too much of the primary vegetation. A large number of secondary forests, with more homogenous species compositions, had grown up in both belts. Most of secondary forests were clustered into the lower montane seasonal rainforest belt, so this group showed a large elevation range.

### 3.2. The Unique Features of the Vertical Vegetation Belts

The vertical vegetation belts of Yarlung Zangbo Grand Canyon National Nature Reserve are similar to Mount Qomolangma [40]. Both of them are one of the most complete vertical vegetation belts in the world. The main reason is that they are located in the south of the Qinghai-Tibet Plain and are affected by the Indian Ocean monsoon. Meanwhile, both have an elevation range of more than 7000 m.

However, Yarlung Zangbo Grand Canyon National Nature Reserve is more humid than the latter because of the major water vapor channel [11,12]. Although the latitude of the former is 29 degrees 37 s, it is 1 degree 38 s higher than that of the latter. Yarlung Zangbo Grand Canyon National Nature Reserve still has the same basic belt as Mount Qomolangma. This is far beyond the latitude where it should be. Therefore, the tropical seasonal rainforest of Yarlung Zangbo Grand Canyon National Nature Reserve is the northernmost tropical seasonal rainforest in the world. Meanwhile, it is also considered to be the northern boundary of the tropical zone in China [15].

The middle montane semi-evergreen broadleaf forest belt is the unique vertical vegetation belt in Yarlung Zangbo Grand Canyon National Nature Reserve. The species of *Cyclobalanopsis* which are dominant trees in the semi-evergreen broadleaf forest, shed all their leaves and then grow new leaves within a month before the rainy season. This way is different from the species of *Cyclobalanopsis,* which were dominant trees in evergreen broadleaf forest belts in East Asian. The latter usually shed their leaves while growing new leaves. The main reason for this difference may be the limitation of rainfall and temperature. In April, which is the end of the dry season, the temperature gradually rises. This is the driest time of the year in Yarlung Zangbo Grand Canyon National Nature Reserve. Deciduous leaves at this time may be an ecological adaptation for the semi-evergreen broadleaf forest to withstand drought stress [39].

### 3.3. Vegetation Conservation

Vegetation classification can improve the conservation planning, monitoring, and management in the reserve by defining clear objects [41]. The knowledge of the vertical vegetation belts and main vegetation types in Yarlung Zangbo Grand Canyon National Nature Reserve are significantly improved by this study. 

The research showed that there are complete vertical vegetation belts and diverse ecosystems. However, the low-elevation primary vegetation, which was seriously disturbed by human activities, has formed a large area of secondary vegetation. At present, only a small primary vegetation remains in valleys and steep places. Therefore, the biodiversity of the region has dropped significantly. As climate change and human activities intensify, the remaining vegetation is also facing a survival crisis [42,43,44,45]. Thus, we recommend that the remaining tropical seasonal rainforest at lower elevations should be protected as early as possible.

Currently, the middle montane semi-evergreen broadleaf forest belt had the highest biodiversity. The main reason is that the conditions of climate here are better and the interference from human activities is less. However, with the improvement of human activity ability, the scope of activity interference has gradually expanded. This belt is suffering from more disturbances than before with grazing, logging, construction, etc. Thus, we recommend that the protection should be enhanced and interference from human activities should be reduced in the middle montane semi-evergreen broadleaf forest belt.

Our study not only showed the vertical vegetation belts and the primary alliance and secondary alliance in each of the vertical belts, but also showed that the main factor affecting vegetation distribution is elevation. As the elevation increases, the average annual temperature gradually decreases. The vegetation type is gradually transitioning from thermophilous lower montane seasonal rainforest belt to cold-tolerating alpine shrubland and meadow belt. Annual rainfall, slope, and aspect were not important as people think in the distribution of vegetation in Yarlung Zangbo Grand Canyon National Nature Reserve [46,47]. By understanding the distribution of vegetation, its composition, and biodiversity patterns, the study provides important theoretical support for the ecological restoration and biodiversity conservation in the reserve [48,49]. 

The adjustment of the reserve and the construction of national parks are being implemented in China. Yarlung Zangbo Grand Canyon National Nature Reserve is recommended as China’s first national park, but there is still a paucity of information about vegetation [10]. Our research provides a base for the management and conservation of these ecosystems in the reserve.

## 4. Materials and Methods

### 4.1. Fieldwork and Data Collection

**Figure 7:**
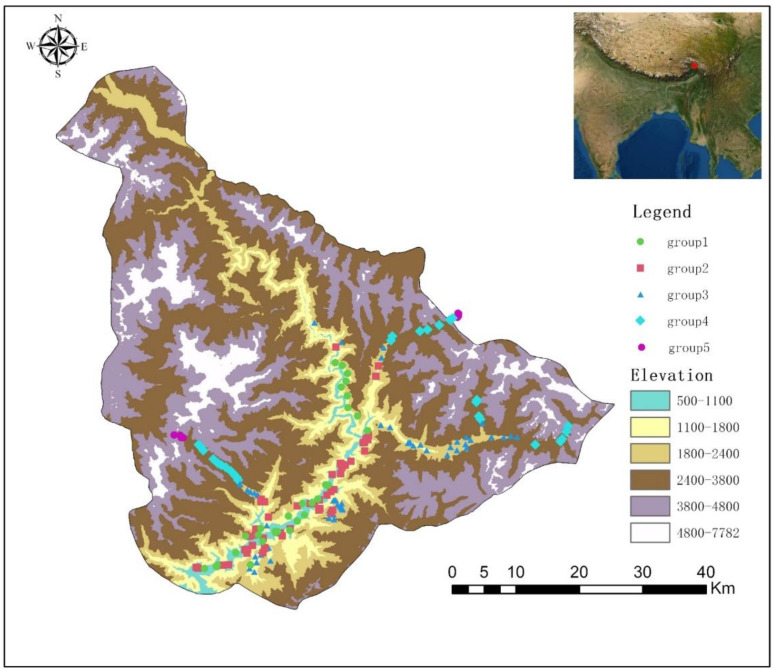
Locations of surveyed plots in Yarlung Zangbo Grand Canyon National Nature Reserve.

Vegetation surveys were conducted from May 2019 to July 2020. Along the elevation gradients of Xirang (550 m)–Duoxiongla (4200 m) and Xiranng (550 m)–Galongla (4300 m), the survey was conducted by every 100 m elevation span (Figure 7). In addition, typical vegetation surveys were conducted in other accessible areas, including plenty of hiking trails. The information of 190 plots were provided in Appendix C. The 20 m × 20 m plot was selected for the forest; the 10 m × 10 m plot was selected for shrubland; the 1 m × 1 m plot was selected for herbaceous vegetation. Density, height, and cover values of each species were recorded, averaged, and changed to their relative values to get the importance value index (IVI) for each species [50,51]. All species were identified according to Flora Xizang, Flora Yunnan, and Flora Reipublicae Popularis Sinicae [38,52,53]. Some species are difficult to identify, which were identified by experts of the corresponding family and genus. Based on the plot coordinates, bioclimatic variables for each study site were extracted from climate grids with a spatial resolution of 30 arc-s [54]. The grid data were downloaded from WorldClim (http://www.worldclim.org (accessed on 1 January 2021)). By using the Spearman correlation coefficient, the correlations between 22 environmental variables were calculated. For variables with spearman correlation coefficients greater than 0.4, the most ecologically important factors were chosen to vegetation analyses. Finally, six variables were reserved. They are: elevation, slope, aspect, annual precipitation (Bio12), precipitation of driest month (Bio14), and precipitation seasonality (Bio15).

### 4.2. Statistical Analyses

The primary data from the field surveys were transformed in a matrix of 190 plots × 1416 species, which were log (x + 1) transformed. Alliances were named according to the vegetation classification system of China [55,56]. The matrix of importance value was subjected to Ward’s method cluster analysis based on Bray–Curtis dissimilarity, by using the stat package [57,58]. The value of fusion level and silhouette width was used to evaluate the rationality of clustering results by using the cluster package [59]. For identifying indicator species significantly associated with each vertical vegetation belt, indicator species analysis was performed by using the indicspecies package [60]. 

To relate the species composition of the accepted groups to environmental variables, Nonmetric Multidimensional Scaling (NMDS) ordination was used based on Bray–Curtis dissimilarity [61]. The lowest stress value was 0.19, which belonged to a two-dimensional configuration. The coordinates of plots were overlaid with the environmental data by using the “envfit” function of the vegan package. The significance of passive vectors was computed using a permutation test with 999 iterations. Bartlett test and Tukey’s honest significant difference test were used to measure the elevation difference between each group in conjunction with ANOVA.

The species richness, Shannon diversity index, Simpson diversity index, and Pielou diversity index were calculated by using the vegan package [61]. The Bartlett test and Kruskal–Wallis test were used to test the diversity differences between five groups. A binomial regression model was used to determine the elevation pattern of species richness by the “lm” function. All analyses were done using R 4.0.3 [58].

We reviewed and summarized previous literature about vertical vegetation belts in Yarlung Zangbo Grand Canyon National Nature Reserve. Based on previous literature and the results of cluster analysis and ordination analysis, we proposed a new scheme for the vertical vegetation belts of the reserve. The new scheme was compared with previous schemes by histogram. Their similarities and differences were discussed.

## 5. Conclusions

We proposed a new division scheme of vertical vegetation belts in Yarlung Zangbo Grand Canyon National Nature Reserve and discussed differences and similarities with previous schemes. The establishment of the semi-evergreen broadleaf forest was supported in the new scheme. The main factor affecting vegetation distribution is elevation. However, the elevation range of the lower montane seasonal rainforest belt and lower montane evergreen broadleaf forest belt was similar. The main reason is that slash-and-burn farming and longtime logging are greatest and most frequent in the region. Thus, the biodiversity of the region has decreased significantly. Meanwhile, the middle montane semi-evergreen broadleaf forests had the highest biodiversity. Therefore, we recommended that tropical seasonal rainforests and semi-evergreen broadleaf forests should be protected as soon as possible. Based on the distribution of vegetation and the condition of biodiversity, local governments can better formulate conservation strategies to optimize conservation efforts and cope with global climate change.

## Figures and Tables

**Table 1:** Relationships of environmental factors and the vertical vegetation belts.

	NMDS1	NMDS2	R^2^	Pr (>r)
Elevation	0.92596	0.37763	0.8681	0.001
Aspect	−0.87558	−0.48307	0.0745	0.001
Slope	−0.09324	−0.99564	0.0287	0.073
Bio12	0.94077	−0.33905	0.0817	0.003
Bio14	−0.39677	0.91792	0.0315	0.058
Bio15	0.0542	−0.99853	0.0728	0.002

**Table 2:** The post-hoc Tukey test for elevation between five groups.

	Diff	Lwr	Upr	P adj
Group 2—Group 1	34.86226	−174.121	243.845	0.990756
Group 3—Group 1	820.0123	647.5891	992.4354	*
Group 4—Group 1	1677.859	1466.588	1889.13	*
Group 5—Group 1	2718.129	2468.598	2967.66	*
Group 3—Group 2	785.15	580.621	989.679	*
Group 4—Group 2	1642.997	1404.8	1881.194	*
Group 5—Group 2	2683.267	2410.561	2955.972	*
Group 4—Group 3	857.8466	650.98	1064.713	*
Group 5—Group 3	1898.117	1652.303	2143.93	*
Group 5—Group 4	1040.27	765.8073	1314.733	*

Abbreviations: Diff = the difference in the observed means; Lwr = the lower end point of the interval; Upr = the upper end point; P adj = the *p*-value after adjustment; * *p* < 0.0001.

**Table 3:** Differences in plant species diversity metrics between five vegetation belts, evaluated by post-hoc Kruskal–Wallis test. Groups with the same letter did not differ significantly at *p* = 0.05.

Cluster Group	Number of Sites	Species Richness per Group (Total)	Species Richness per Site (Mean)	Shannon Diversity Index, per Site (Mean)	Simpson Diversity Index, per Site (Mean)	Pielou Diversity Index, per Site (Mean)
group 1	53	618	42.79 ^a^	3.16 ^a^	0.91 ^a^	0.86 ^a^
group 2	31	423	38.74 ^b^	3.08 ^b^	0.91 ^b^	0.85 ^b^
group 3	60	823	46.43 ^c^	3.26 ^c^	0.92 ^c^	0.86 ^c^
group 4	28	459	39.32 ^d^	3.03 ^d^	0.91 ^d^	0.83 ^d^
group 5	18	185	7.02 ^e^	2.72 ^e^	0.89 ^e^	0.86 ^e^

**Figure A1:**
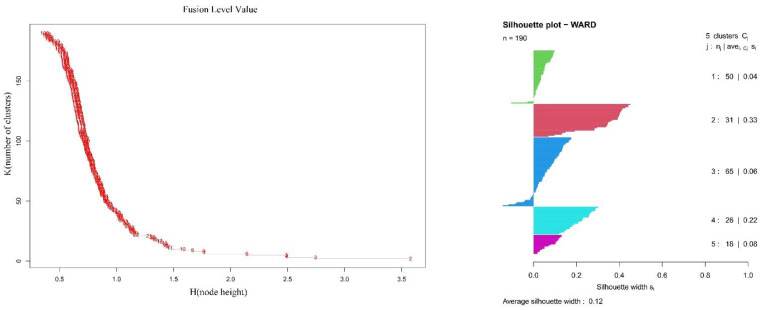
The plots of fusion level value and silhouette width.

## Data Availability

Not applicable.

## Appendix B

**Table A1:** The top 20 indicator species in five vertical vegetation belts.

Species Name	Group1		Group2		Group3		Group4		Group5	
	IndVal	*p*	IndVal	*p*	IndVal	*p*	IndVal	*p*	IndVal	*p*
*Altingia excelsa*	0.411	0.001								
*Impatiens stenantha*	0.368	0.001								
*Phrynium placentarium*	0.339	0.001								
*Impatiens namchabarwensis*	0.314	0.002								
*Sambucus ebulus*	0.298	0.002								
*Mussaenda decipiensis*	0.291	0.004								
*Terminalia myriocarpa*	0.284	0.006								
*Boehmeria macrophylla* var. *rotundifolia*	0.279	0.004								
*Lagerstroemia minuticarpa*	0.276	0.008								
*Blumea balsamifera*	0.275	0.004								
*Cordia dichotoma*	0.26	0.006								
*Macaranga denticulata*	0.258	0.008								
*Trachelospermum jasminoides*	0.253	0.007								
*Dichrocephala benthamii*	0.252	0.014								
*Polygonum chinense*	0.245	0.007								
*Gynostemma pentaphyllum*	0.238	0.018								
*Poikilospermum suaveolens*	0.238	0.02								
*Equisetum diffusum*	0.231	0.035								
*Chrysopogon aciculatus*	0.225	0.032								
*Syngonium podophyllum*	0.224	0.029								
*Castanopsis indica*			0.901	0.001						
*Glochidion hirsutum*			0.462	0.001						
*Oplismenus undulatifolius*			0.404	0.001						
*Triumfetta cana*			0.368	0.001						
*Pteris cretica* var. *nervosa*			0.333	0.001						
*Desmodium sequax*			0.32	0.001						
*Colocasia antiquorum*			0.314	0.003						
*Strobilanthes dimorphotricha*			0.314	0.001						
*Polygonum capitatum*			0.307	0.002						
*Saurauia napaulensis*			0.296	0.002						
*Elatostema acuminatum*			0.287	0.009						
*Circaea cordata*			0.287	0.009						
*Chirita pumila*			0.287	0.013						
*Dioscorea hispida*			0.287	0.009						
*Solena amplexicaulis*			0.287	0.006						
*Hedyotis scandens*			0.287	0.004						
*Wallichia disticha*			0.257	0.006						
*Ophiorrhiza mungos*			0.253	0.019						
*Impatiens arguta*			0.25	0.017						
*Engelhardtia spicata*			0.249	0.009						
*Cyclobalanopsis lamellosa*					0.571	0.001				
*Exbucklandia populnea*					0.378	0.001				
*Cyclobalanopsis kiukiangensis*					0.367	0.001				
*Damnacanthus indicus*					0.361	0.001				
*Pholidota articulata*					0.352	0.001				
*Ficus sarmentosa*					0.348	0.001				
*Disporum bodinieri*					0.342	0.001				
*Arisaema concinum*					0.341	0.001				
*Ainsliaea latifolia*					0.315	0.001				
*Vaccinium kingdom-wardii*					0.31	0.001				
*Myrsine semiserrata*					0.306	0.002				
*Pinus bhutanica*					0.29	0.003				
*Remusatia vivipara*					0.289	0.003				
*Toxicodendron hookeri* var. *microcarpum*					0.288	0.001				
*Ilex wilsonii*					0.288	0.002				
*Viburnum cylindricum*					0.287	0.001				
*Betula cylindrostachya*					0.277	0.004				
*Pyrrosia lanceolata*					0.269	0.004				
*Tupistra aurantiaca*					0.268	0.005				
*Taxillus dalavayi*					0.267	0.004				
*Tsuga dumosa*							0.717	0.001		
*Abies delavayi* var. *motuoensis*							0.69	0.001		
*Acanthopanax evodiaefolius*							0.49	0.001		
*Ribes glaciale*							0.483	0.001		
*Lindera obtusiloba*							0.443	0.001		
*Pilea notata*							0.434	0.001		
*Acer campbellii*							0.426	0.001		
*Synotis alata*							0.375	0.001		
*Circaea alpina*							0.37	0.001		
*Smilacina fusca*							0.359	0.001		
*Lonicera tangutica*							0.353	0.002		
*Lunathyrium medogense*							0.353	0.001		
*Oxalis acetosella* subsp. *Leucolepis*							0.348	0.001		
*Gaultheria trichophylla*							0.347	0.001		
*Dysosma tsayuensis*							0.345	0.003		
*Acer* sp.							0.335	0.001		
*Arisaema rhizomatum*							0.333	0.002		
*Arisaema biauriculatum*							0.328	0.001		
*Mahonia fortunei*							0.32	0.003		
*Euonymus alatus*							0.316	0.001		
*Pleurospermum angelicoides*									0.534	0.001
*Dryopteris barbigera*									0.511	0.001
*Viola biflora*									0.486	0.001
*Athyrium attenuatum*									0.482	0.001
*Geranium polyanthes*									0.474	0.001
*Cardamine macrophylla*									0.469	0.001
*Pedicularis lineata*									0.467	0.001
*Polygonum polystachyum*									0.466	0.001
*Rosa sericea*									0.461	0.001
*Rhododendron viridescens*									0.457	0.001
*Ribes orientale*									0.456	0.001
*Polygonum viviparum*									0.445	0.001
*Cirsium eriophoroides*									0.445	0.001
*Gaultheria dolichopoda*									0.444	0.001
*Anaphalis margaritacea* var. *japonica*									0.44	0.001
*Lonicera angustifolia* var. *myrtillus*									0.425	0.001
*Rhodiola rosea*									0.416	0.001
*Saxifraga* sp.									0.416	0.001
*Polygonatum verticillatum*									0.408	0.001
*Senecio lingianas*									0.396	0.001

## Appendix C

**Table A2:** The information of 190 plots.

Plot	Elevation	Longitude	Latitude	Aspect	Slope
CaIn32	635	95.051	29.185	153.43	25.84
CaIn21	718	95.054	29.188	286.02	39.18
made3	759	95.084	29.188	191.59	18.35
CaIn10	815	95.103	29.215	247.61	20.95
lixi2	664	95.004	29.182	202.69	13.94
cain	867	95.021	29.181	136.25	28.20
made2	1518	95.131	29.256	187.35	38.00
CaIn6	1557	95.129	29.253	7.41	43.13
fise5	1415	95.14	29.252	157.28	21.22
oxpa	1472	95.156	29.269	116.68	42.57
fiau	1152	95.18	29.331	292.03	20.68
expo8	2079	95.348	29.308	193.09	20.19
pana	2029	95.354	29.303	187.59	7.19
dibu	1615	95.179	29.224	85.31	14.30
expi	1621	95.182	29.228	270.00	28.07
cyki6	1479	95.153	29.206	214.17	22.19
CaIn5	1677	95.14	29.187	11.85	19.02
expo7	1843	95.168	29.21	334.33	19.59
cyla18	1863	95.168	29.21	317.68	20.68
cyki1	1644	95.189	29.225	227.60	14.54
expo6	1823	95.169	29.219	2.94	36.13
sapo4	1679	95.178	29.226	90.90	28.07
lipu	1018	95.132	29.214	321.74	28.89
alex19	669	95.127	29.222	310.33	16.15
temy4	814	95.147	29.232	350.39	15.35
alex8	1200	95.155	29.255	119.05	34.46
temy3	595	94.999	29.183	203.19	31.08
CaIn4	631	95.069	29.187	166.07	28.21
CaIn3	895	95.174	29.24	320.42	43.08
alex7	853	95.134	29.222	343.30	38.06
alex6	859	95.175	29.247	234.59	22.99
alex5	1648	95.174	29.214	52.25	18.09
ensp2	722	95.162	29.255	123.08	33.28
CaIn2	758	95.169	29.27	113.19	15.93
alex4	1115	95.206	29.263	60.26	3.84
CaIn1	1127	95.207	29.26	326.53	16.41
CaIn31	1063	95.213	29.262	325.30	7.51
CaIn30	1063	95.213	29.262	325.30	7.51
CaIn29	789	95.246	29.268	38.66	3.05
CaIn28	865	95.264	29.285	320.35	10.72
tsdu11	2458	95.099	29.395	127.47	17.48
tsdu8	2361	95.103	29.391	233.13	24.62
cyts	2377	95.108	29.387	232.98	34.15
cyki5	2308	95.111	29.381	229.35	31.82
cyla9	2202	95.117	29.373	180.00	15.37
cyla8	2189	95.117	29.372	58.32	12.95
cyla7	1990	95.135	29.358	70.11	11.76
cyki4	1847	95.144	29.354	170.28	37.42
expo5	1958	95.153	29.351	40.49	12.65
cyki3	1877	95.162	29.347	82.21	21.77
fise4	1139	95.179	29.331	127.14	9.79
cyla6	1224	95.172	29.329	278.13	31.74
CaIn27	842	95.244	29.264	62.30	20.63
CaIn26	820	95.284	29.299	278.24	31.40
CaIn25	820	95.279	29.325	339.53	16.59
CaIn24	887	95.382	29.414	171.09	19.29
figl	797	95.358	29.39	132.31	20.68
alex3	1407	95.225	29.252	18.43	4.52
alex2	1487	95.226	29.258	136.24	15.16
cahy2	1393	95.22	29.259	306.20	30.04
CaIn23	1216	95.341	29.367	132.43	21.56
CaIn22	856	95.347	29.366	115.49	16.70
CaIn20	746	95.319	29.335	147.45	27.42
CaIn19	802	95.304	29.326	161.00	15.04
fise3	1125	95.265	29.32	65.67	35.67
cete	835	95.299	29.325	146.51	17.99
lixi1	1830	95.19	29.296	188.13	32.95
expo4	1617	95.184	29.277	61.93	15.81
CaIn18	1407	95.242	29.297	202.59	31.72
lami	896	95.393	29.411	1.81	21.60
temy2	849	95.387	29.41	347.59	23.10
CaIn17	1014	95.384	29.406	316.12	16.72
alex1	952	95.38	29.392	344.74	18.39
cyki2	1998	95.381	29.323	108.68	31.44
cyki8	2107	95.37	29.32	156.25	6.49
cyla5	1984	95.38	29.313	60.12	12.72
cyki7	2044	95.377	29.317	49.40	8.74
CaIn16	2043	95.365	29.332	5.71	14.10
cyla4	2136	95.359	29.298	303.99	12.19
rhde	1940	95.358	29.309	17.56	19.04
coca2	1843	95.36	29.331	257.30	25.36
alsh	1084	95.362	29.356	188.91	40.70
alex18	923	95.353	29.351	174.17	39.38
alca	2435	95.36	29.287	148.67	12.64
cyla1	2303	95.347	29.291	23.59	38.34
ospa	1308	95.324	29.313	322.22	22.86
alex17	1365	95.322	29.309	297.83	22.75
sapo3	824	95.323	29.332	301.90	27.48
alex16	754	95.31	29.324	326.04	13.82
fise2	1261	95.343	29.343	287.14	25.01
pibh6	1579	95.184	29.224	333.43	2.13
made1	832	95.383	29.413	153.71	37.64
alex15	947	95.38	29.416	190.00	26.72
alex14	895	95.408	29.42	261.86	34.12
pibh5	1997	95.349	29.303	62.10	4.58
coca1	1812	95.359	29.33	260.86	25.29
pibh4	1814	95.193	29.195	309.80	11.04
alex13	1340	95.448	29.471	223.38	22.71
caec2	1434	95.453	29.468	236.50	34.19
alex12	1407	95.455	29.478	305.70	18.18
CaIn15	1343	95.453	29.489	333.43	14.62
cyla3	2028	95.487	29.501	125.44	31.11
pibh3	1528	95.512	29.496	43.85	16.41
expo3	1657	95.558	29.458	28.44	12.81
expo2	1798	95.571	29.452	357.66	42.66
caec1	1691	95.563	29.46	199.35	18.09
deti	1797	95.444	29.442	338.11	45.16
muba	876	95.444	29.462	347.08	24.98
cahy1	1651	95.558	29.459	210.96	17.52
lixy	1785	95.59	29.454	160.01	5.57
pibh2	1750	95.606	29.441	298.30	7.01
saps2	1977	95.66	29.429	346.84	24.60
pibh1	2155	95.668	29.447	180.83	40.78
expo1	2001	95.685	29.439	323.23	24.34
jusi3	2336	95.686	29.459	180.00	4.29
saps1	2151	95.721	29.462	159.37	22.49
jusi2	2641	95.71	29.469	128.75	20.77
powi	2192	95.776	29.468	189.36	21.02
saps4	2821	95.739	29.553	296.56	8.48
saps3	2788	95.737	29.548	218.29	5.76
tsdu7	2621	95.743	29.515	290.46	16.59
tsdu6	2605	95.747	29.508	198.43	15.46
cyla2	2492	95.753	29.5	264.55	9.97
temy1	826	95.441	29.461	296.38	31.35
rhsa3	3498	94.971	29.481	81.53	11.19
rhsa2	3498	94.971	29.481	81.53	11.19
rhsa1	3498	94.971	29.481	81.53	11.19
safl3	3613	94.966	29.481	74.95	21.86
safl2	3613	94.966	29.481	74.95	21.86
safl1	3613	94.966	29.481	74.95	21.86
rhch	4255	94.947	29.487	9.32	15.79
sare4	3834	94.963	29.486	144.21	48.74
sare3	3834	94.963	29.486	144.21	48.74
sare2	3834	94.963	29.486	144.21	48.74
abmo10	3233	95.009	29.463	237.65	16.48
abmo8	3186	95.014	29.458	231.34	36.76
abmo7	2969	95.021	29.45	231.65	19.59
ensp1	2023	95.493	29.655	124.87	18.53
sapo2	1770	95.485	29.637	258.94	20.27
cyla17	2229	95.499	29.678	106.69	27.56
tsdu5	2454	95.518	29.694	136.34	26.61
tsdu4	2602	95.522	29.703	253.55	24.52
tsdu3	2813	95.594	29.713	187.12	15.04
abmo6	3007	95.614	29.716	207.47	6.70
abmo5	3213	95.645	29.726	203.62	8.28
fise1	1576	95.478	29.613	96.43	29.14
abmo4	3424	95.673	29.737	154.23	15.02
abmo3	3589	95.681	29.741	181.07	41.72
jusi1	3712	95.696	29.753	158.42	43.92
CaIn14	1721	95.138	29.179	336.80	1.82
CaIn13	1551	95.15	29.171	71.57	12.62
CaIn12	1540	95.156	29.193	242.35	30.65
cyla16	2048	95.384	29.309	7.43	5.52
cyla15	2034	95.382	29.31	1.00	8.53
cyla14	2280	95.71	29.444	335.44	20.35
cyla13	2218	95.708	29.453	305.21	9.84
abmo2	3273	95.976	29.488	51.73	49.16
abmo1	3151	95.971	29.475	116.80	24.36
abmo9	2918	95.959	29.459	111.50	16.46
tsdu2	2827	95.955	29.453	156.54	13.53
tsdu1	2626	95.888	29.447	220.03	7.75
tsdu10	2519	95.843	29.466	194.56	28.66
tsdu9	2404	95.827	29.469	191.30	9.65
cyla12	2288	95.808	29.467	198.43	18.23
alex11	1152	95.374	29.682	223.31	11.33
sapo1	1723	95.372	29.646	296.20	41.27
cace	1569	95.391	29.639	155.19	28.64
rhme12	4219	95.696	29.753	136.63	31.75
rhme1	4130	95.698	29.752	138.57	10.69
saan2	4000	95.698	29.747	191.18	36.14
saan1	3907	95.695	29.746	208.51	37.53
sare1	3789	95.695	29.744	187.76	36.50
cyla11	2134	95.32	29.738	258.56	36.33
cyla10	2462	95.389	29.692	225.60	28.98
alex10	1473	95.404	29.621	287.96	17.95
CaIn11	1623	95.399	29.603	345.59	38.21
alex9	1561	95.387	29.59	228.86	41.15
CaIn9	1505	95.402	29.569	214.00	39.84
CaIn8	1929	95.402	29.542	343.94	48.85
CaIn7	1707	95.427	29.523	247.68	40.55
povi	4255	94.947	29.487	9.32	15.79
bepu	4255	94.947	29.487	9.32	15.79
amhi	1253	95.17	29.331	222.39	14.54
fise6	1314	95.171	29.337	247.14	28.68
expo9	1754	95.163	29.345	132.31	37.05
tsdu12	2875	95.047	29.43	174.55	9.97
tsdu13	2764	95.059	29.42	173.89	33.67
tsdu14	2703	95.067	29.416	180.00	15.37
tsdu15	2603	95.079	29.411	121.60	7.25
tsdu16	2486	95.092	29.401	113.74	6.49
